# Emerging role of exosome-mediated intercellular communication in vascular remodeling

**DOI:** 10.18632/oncotarget.14878

**Published:** 2017-01-28

**Authors:** Sheng-An Su, Yao Xie, Zurong Fu, Yaping Wang, Jian-An Wang, Meixiang Xiang

**Affiliations:** ^1^ Department of Cardiology, Cardiovascular Key Lab of Zhejiang Province, Second Affiliated Hospital, Zhejiang University College of Medicine, Hang Zhou, Zhejiang, P.R. China; ^2^ Cardiovascular Division, Kings College London BHF Center, London, United Kingdom

**Keywords:** exosome, vascular remodeling, endothelial function, atherosclerosis, vascular repair

## Abstract

Vascular remodeling refers to the alternations of function and structure in vasculature. A complex autocrine/paracrine set of cellular interaction is involved in vascular remodeling. Exosome, a newly identified natural nanocarrier and intercellular messenger, plays a pivotal role in regulating cell-to-cell communication. Exosome emerges as an important mediator in the process of vascular remodeling, showing the most prognostic and therapeutic potent in vascular diseases. Benefiting from exosomal trafficking, the vasculature can not only maintain its function and structure in physiological condition, but also adapt itself in pathological status. In this review, we will represent the roles of exosomes in angiogenesis, endothelial function and cardiac regeneration. In addition, greatly depending on the pathophysiological status of donor cells and peripheral micro-circumstance, the exosomal content could alter, which makes exosomes exhibit pleiotropic effects in vascular diseases. Hence, the diverse effects of exosomes in vascular diseases including atherosclerosis, neointima formation and vascular repair, primary hypertension, pulmonary artery hypertension, and aortic aneurysm will be discussed. Finally, the translational appliances targeting exosomes will be concluded by providing updated applications of engineered exosomes in clinic.

## INTRODUCTION

Vascular wall is an active and integrated organ, consisting of endothelial cells, smooth muscle cells, fibroblasts and extracellular matrix. The vasculature is sensitive to various stimuli, many of which could contribute to physiopathologic changes. After going through a complex autocrine/paracrine set of cellular interaction in response to different stimulus, the vasculature eventually adapts itself to the local environment by integrating the signals, and producing mediators which in turn influence the function and structure of itself. This dynamic process of structural alterations is defined as “vascular remodeling” [1]. Vascular remodeling mainly involves four processes—cell growth, cell death, cell migration, and production or degradation of extracellular matrix [2]. It is not only the hallmark of vascular diseases such as atherosclerosis, vessel restenosis, primary hypertension, pulmonary artery hypertension and aortic aneurysm, but also the maintenance of vascular function and structure after injury.

Intercellular communication, a key process in vascular remodeling, is originally believed to be achieved by either direct cell-to-cell contact or paracrine effects. However, a third mechanism secreting extracellular vesicles was recently reported. Currently, a major concern in the literature of extracellular vesicles is that there is no broad consensus of the nomenclature [3]. Multifarious names have been used such as exosomes, microparticles, microvesicles, ectosomes, apoptotic bodies, etc. In this review, we will use the nomenclature “extracellular vesicles” which was proposed by Chistiakov et al. [4]. Distinguished by the size, lipid composition, marker proteins, and mechanisms of formation and discharge, extracellular vesicles include three types of plasma membrane-shed vesicles— exosomes, microvesicles and apoptotic bodies.

**Figure 1:**
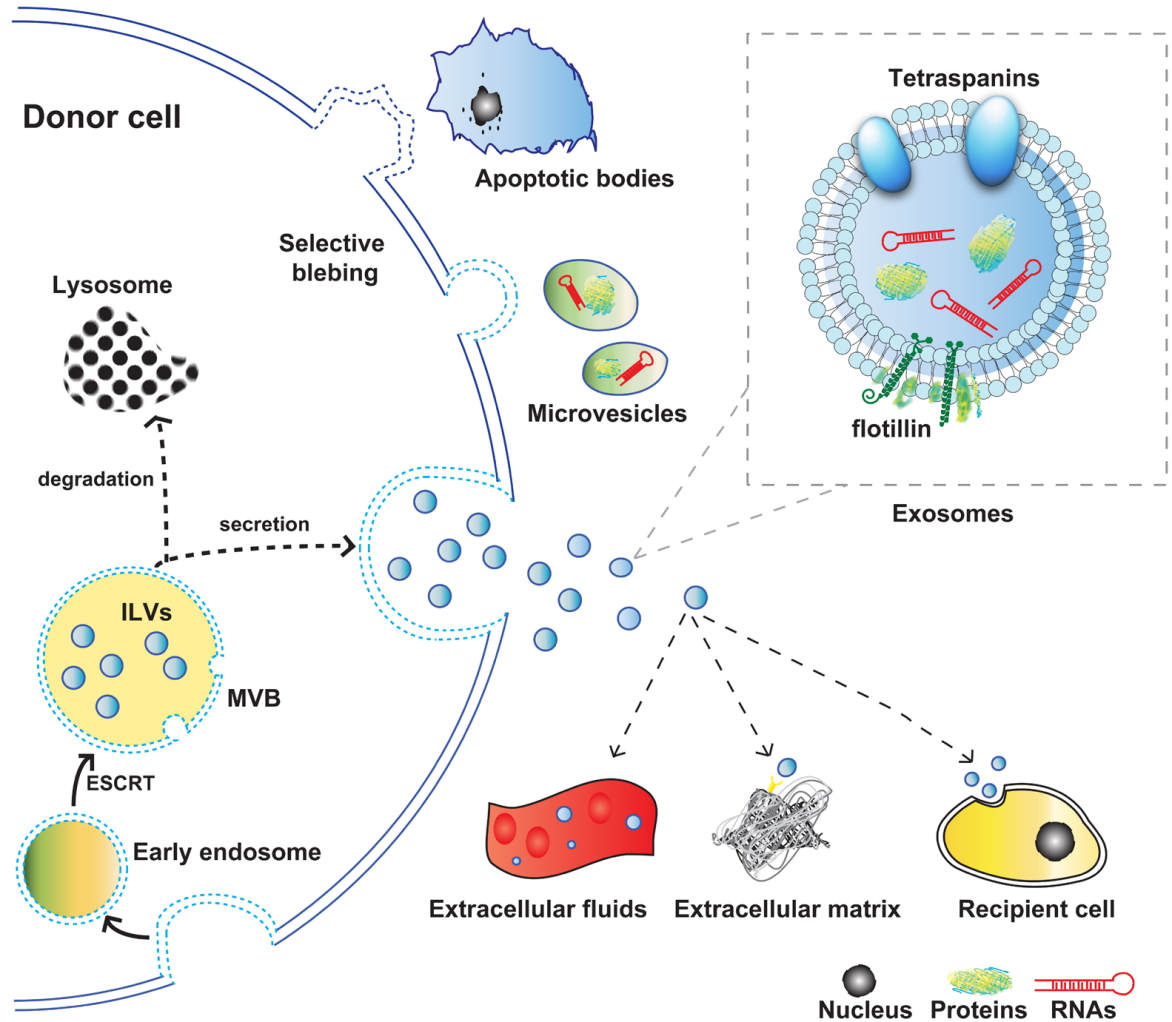
Schematic representation of the origin, release, and structure of exosomes Abbreviations used: MVB, multivesicular body; ILV, intraluminal vesicle.

Exosomes have the smallest size which is normally between 30-100nm, while microvesicles are generally between 100nm and 1μm, and apoptotic bodies share the biggest size more than 1μm. Exosomes are generated by endosomal pathway leading to the inward budding of multivesicular bodies (MVBs). Under the stimuli of various physical and/or chemical factors, the plasma membrane inward buds in the ceramide-triggered budding mechanism, and produces early endosomes (EEs) [5]. The further inward budding of the late endosomal membrane induces multivesicular bodies named MVBs. This process could be directed by the machinery of endosomal sorting complex required for transport (ESCRT), resulting in the progressive accumulation of intraluminal vesicles (ILVs) within MVBs. Intracellular MVBs exhibit dynamic alterations of the components in response to different pathologic or physiologic cellular status. MVBs can either traffic to lysosomes where they are eventually degraded by proteasomes, or secret ILVs which contain transmembrane proteins and other functional cytosolic components like miRNA and mRNA into extracellular space upon fusion with the plasma membrane. The released ILVs are referred as “exosomes” [6, 7]. The lipid bilayer of exosome is mainly composed by phosphatidylcholine (PC), ganglioside GM3, phosphatidyl ethanolamine (PE), sphingomyelin (SM), cholesterol, and lipid rafts which is a cholesterol-rich membrane microdomain [8]. The multiple lipid composition contributes to both an integrated structure and signal transmission of exosomes [9],[10]. In addition, exosomes are enriched in several specific protein markers such as tetraspanins CD63, CD81, CD9, and CD82, flotillin and tumor susceptibility gene 101 (TSG101), which make exosomes distinguishable from other extracellular vesicles [4]. (Figure 1)

Briefly, the other two extracellular vesicles—apoptotic bodies and microvesicles, also named microparticles, are formed through direct plasma membrane blebbing with the involvement of cytoskeleton rearrangement. Their lipid compositions of cholesterol and high phosphatidylserine exposure also differ from exosomes. Besides, microvesicles have irregular shape and density without any tetraspanins in the surface. On contrary, apoptotic bodies are activated during the late phase of apoptosis, within the package of cracked cell organelles and fragmented nuclear components [11, 12].

Extracellular vesicles serve as intercellular messengers. Especially exosomes are not only emerging as significant mediators in the process of vascular remodeling, but also showing their potential in prognostic and therapeutic applications in vascular diseases. A growing number of publications have explored the role of exosomes in vascular remodeling, whereas, surprisingly, few have been reviewed so far. Therefore, we provide an overview based on current knowledge about exosomes during the pathologic vascular remodeling. We will also summarize recent promising translational applications of engineered exosomes in clinic.

## ANGIOGENESIS, ENDOTHELIAL FUNCTION AND REGENERATION

Angiogenesis and endothelial regeneration both of which related to the endothelial proliferation, migration and survival, are highlighted in cardiovascular and cancer research. Major function of endothelial cells in angiogenesis requires exosomes. Sheldon et al. revealed that the exosomes from endothelial cells regulate the process of neovascularization. Exosomes from natural endothelial cells with Delta-like 4 (Dll4), a ligand for Notch signaling, could be internalized by the adjacent endothelial cells. The subsequent inhibition of Notch signaling eventually switched the endothelial cell phenotype to tip cells. This alternation resulted in an increase in vessel density *in vitro* and numbers of branching *in vivo* [13]. However, controversially, the Dll4-containing exosomes in the fully-formed tip cells seemed to induce capillary sprout retraction [14]. Thereafter, other two studies also demonstrated that miR-214, an angiogenic miRNA, and Angiopoietin-2 which is the principal ligand of Tie2 receptor involved in the regulation of vascular integrity, are incorporated into exosomes in natural endothelial cells, leading to endothelial cell proliferation *in vitro* and angiogenesis *in vivo* [15, 16].

Thanks to its multiple cell origins and biochemical properties, the role of exosomes in tumor angiogenesis is diverse. Overall, the exosomes derived from the majority of tumor cell types exhibit a proangiogenic phenotype in tumor angiogenesis. These malignant cell-derived exosomes could be enriched with various proangiogenic factors such as tetraspanin Tspan8, developmental endothelial locus-1, miR-210, miR-135b, etc [17–20]. They also contained growth factors like fibroblast growth factor 2 to promote the tumor invasion, and pro-metastatic miRNA like miR-105 to destroy the distant vascular permeability for tumor metastasis [21, 22]. Therefore, exosome could possibly be a good biomarker of tumor that predicts the cancers without clinical symptoms.

Meanwhile, the angiogenic role of exosomes in cardiology is still ambiguous. Cardiomyocytes have an intimate anatomical relationship with cardiac endothelial cells. However, the studies about the crosstalk between cardiomyocytes and endothelial cells are limited. Garcia et al. discovered that with glucose deprivation, exosomes containing an enrichment of proangiogenic miR-17, miR-19, miR-20a, miR-30c and miR-126 from cardiomyocytes could promote the migration and proliferation of endothelial cells [23]. Further study identified that the cardiomyocyte-derived exosomes with starvation were also loaded with functional glucose transporters and glycolytic enzymes, leading to increased glucose uptake, glycolytic activity and pyruvate production in recipient endothelial cells [24]. Thus, the exosome trafficking between cadiomyocytes and endothelial cells established a metabolic regulation, potentially indicating the induction of local neovascularization under acute stress. Interestingly, in type2 diabetic rat model, the diabetic cardiomyocyte-derived exosomes inhibited endothelial migration and proliferation. The high expression of miR-320 and low expression of miR-126 in the exosomes were linked to their anti-angiogenic effect [25]. Overall, exosomes emerged as a regulatory factor that influences the endothelial function and alters the cardiac microenvironment.

Additionally, the pro-angiogenic effect after myocardial infarction was also discovered in stem cell-derived exosomes. After myocardial infarction injury, multisource-derived stem cells such as embryonic stem cells, mesenchymal stem cells, hematopoietic stem cells and cardiac progenitor cells were capable of stimulating neovascularization and promoting cardiac repair through exosomal transmission [26–29]. The reported mechanisms mainly focused on the regulatory role of the transferred miRNAs such as miR-126, miR-294, etc [29, 30]. Recently, Quesenberry et al. have summarized that the extracellular RNA-carrying vesicles including exosomes could mediate cell fate alterations in the repair of tissue injury. During the process of repair, vesicle-mediated exchange of information could be bidirectional between stem cells and injured cells. Tissue injured cells could induce gene expression and differentiation decisions in the stem cells via vesicles. Conversely, stem cell-derived vesicles could reprogram injured cells by activating regenerative mechanisms. Particularly, the encapsulated RNAs including mRNA, miRNA, siRNA in vesicles may induce phenotypic and functional changes of recipient cells [31]. However, the detailed mechanism that how the exosomal signals alter cell function or reprogram targeted cells in tissue repair is still ambiguous and has yet to be further elucidated.

## ROLE OF EXOSOMES IN VASCULAR DISEASE

Exosome and its messenger role are recently regarded as a critical factor in vascular remodeling, which could be a promising biomarker in the clinic. To elucidate the role of exosomes in angiogenesis, many progresses have been made over past few years.

**Table 1:** Summary of studies reporting exosome-mediated intercellular communication in the process of vascular remodeling and vascular diseases

	Donor cell	Target cell	Contents delievered	Effects
***Exosomal transfer in angiogenesis, endothelial function and regeneration***				
	EC	EC	Dll4, miR-214, Angiopoietin-2	Regulating neovascularzation [13, 15, 16]
	Malignant cell	EC	Tspan8, developmental endothelial locus-1, miR-210, miR-135b, FGF2, miR-105, etc	Tumor angiogenesis [17–22]
	CM	EC	miRNA-17, miR-19, miR-20a, miR-30c, miR-126, functional glucose transporters, glycolytic enzymes	Promoting EC functions and exhibiting a metabolic regulation on EC in starving status [23, 24]
	CM	EC	miR-320	Anti-angiogenic effect in high glucose environment [25]
	Stem cell	EC	miR-126, miR-194, etc	Promoting cardiac repair after myocardial infarction [26–31]
***Exosomal transfer in atherosclerosis***				
	EC	Monocyte	HSP70	Enhanced monocyte adhesion [34]
	EC	VSMC	miR-143/145	Targeting on transformation of VSMC phenotype to alleviate the atherosclerotic plaque [35]
	VSMC	Atherosclerotic plaques	miR-221/222	Pro-atherosclerotic effect from the diabetic VSMC [37]
	VSMC	VSMC	Calcium-binding and extracellular matrix proteins.	Increasing calcification of VSMCs in response to environmental calcium stress [38]
	leukocyte	Monocyte, EC	SRY	Increasing adherence of monocytes and EC, accelerating atherosclerosis [40]
	Monocyte and monocyte cell lines	Monocyte	HSP70, IL-1β, Gal-3	Cell activation and differentiation towards macrophage [42, 43]
	Macrophage	EC	integrin-1	Suppressing EC migration [46]
	Monocyte/macrophage	Circulation	Gal-3, TRX-1/PRDX-1	Closely associated with atherosclerosis severity [43, 47]
	Macrophage	VSMC	S100A9, Annexin V	Accelerating microcalcification [48, 49]
	CD4+ T cell	Monocyte	cholesterol	Enhancing cholesterol accumulation [51]
	Mast cell	EC	PAI-1	Endothelial cell dysfunction and resulting in procoagulant states [54]
	Plt	Plt, Macrophage	Ubiquitinated proteins	Suppressing ex-vivo platelet aggregation, reducing adhesion to microfluidic flow, reducing CD36-dependent lipid loading on macrophage, exhibiting an anti-thrombogenesis effect [59]
	Plt	Unclear	miR-223	Potential pro-atherosclerotic effect [60–62]
***Exosomal transfer in neointima formation and vascular repair***				
	Unclear	EPC	miR-15/16	Impairing the early EPCs and positively correlated with restenosis after post-revascularization of critical limb ischemia patients [65]
***Exosomal transfer in primary hypertension***				
	CM	CM, skeletal myocytes	AT1R	Improving sensitivity to RAS of the target cells and adapting them to the fluctuation of blood pressure under cardiac pressure overload [68]
	Macrophage Or hypertensive serum	EC	Unclear	Endothelial damage through p38-MAPK activation and ICAM-1 expression [69]
	Urinary	Unclear	sodium transporters, miR-615, miR-211, etc	Correlate with the progression of hypertension [71–76]
***Exosomal transfer in pulmonary arterial hypertension***				
	VSMC	EC	miR-143	Promoting EC migration and angiogenesis thus involving in the pathogenesis of PAH [78]
	MSC	EC	Unclear	Suppression of STAT3 and upregulation of the miR-17 superfamily in EC, thus making a cytoprotective action in pulmonary hypertension [79]
***Exosomal transfer in aortic aneurysm***				
	Serum from AAA patients	Unclear	PF-4, ferritin light chain, HSP60, CRP	Potential pathogenetic role in pathogenesis of AAA [82]
	MSC	Macrophage, VSMC	Unclear	Anti-inflammatory effect [88]

Abbreviations used: EC, endothelial cell; CM, cardiomyocyte; VSMC, vascular smooth muscle cell; Plt, Platelet; EPC, endothelial progenitor cell; MSC, mesenchymal stromal cell; AAA, abdominal aortic aneurysm.

### Atherosclerosis

The dysfunction of endothelial cells, the recruitment and proliferation of VSMCs from media to intimal layer, and the infiltration of inflammatory cells are key factors of atherosclerosis [32]. Exosome is employed as an important transporter to regulate the communications among the involved cells in atherosclerosis.

#### Endothelial cell-derived exosomes in atherosclerosis

Endothelial dysfunction is the critical first step towards atherosclerotic plaque formation. Endothelial dysfunction is triggered by the deposition of oxidized low-density lipoprotein (ox-LDL) or the predisposition of hyperlipidemia, hyperglycemia, and smoking [33]. Several studies recently focused on exosomal trafficking in atherosclerosis. In rat endothelial cells, exogenous ox-LDL induced exosomal heat shock protein 70 (HSP70) secretion, resulting in monocyte adhesion to endothelial cells [34]. Interestingly, the exosomes from endothelial cells which overexpressed the shear-responsive transcription factor Kruppel-like factor 2 (KLF2), were shown to alleviate the atherosclerotic plaque by transfusion into ApoE^−/−^ mice. Further *in-vitro* experiment confirmed that the atheroprotective effect was achieved by endothelial cell-derived exosomes containing miR143/145, a downstream production of KLF2 and also the booster to transform the phenotype of VSMCs [35]. Nevertheless, Climent et al. discovered that in the co-cultured system, miR143/145 cluster were transferred from VSMC to EC through tunneling nanotubes instead of exosomes, which finally promoted angiogenesis [36] In short, KLF2 and miR143/145 cluster exerted the atheroprotective role through a vesicle trafficking route of communication between EC and VSMC. However, a lot more work is heavily required to identify the content-specific exosomes released by endothelial cells and the effect of exosomes on atherosclerosis.

#### VSMC-derived exosomes in atherosclerosis

The recruitment and proliferation of VSMC depend on its transformation from “contractile” state to “synthetic” state. VSMCs not only sense the stimulations from the circumstance to start conversion, but also communicate with other cells. Recently, the role of VSMC-derived exosomes in intercellular transport was clarified by Lightell et al. In a diabetic mouse model, exosomes derived from VSMCs exhibited more pro-atherosclerotic miR-221/222 than that in non-diabetic mice. Administration of the diabetic exosomes to ApoE^−/−^ mice resulted in the aggravation of atherosclerotic lesion [37]. In cultured human VSMC calcification model, which mimicked vascular calcification of atherosclerosis, the secreted matrix vesicles were identified as exosomes, which were enriched in calcium-binding and extracellular matrix proteins. The calcifying exosomes could be triggered by tumor necrosis factor-α and platelet derived growth factor-BB, leading to the increased calcification of VSMCs [38]. Interestingly, during the process that VSMCs underwent from the quiescent “contractile” to the active “synthetic” and then to the “calcifying” statue, the contents of exosomes altered. By applying the technology of proteomic analysis, the exosomes released from synthetic VSMCs were rich in calcification inhibitors such as fetuin-A and other cargoes related to adhesion and migration. Afterwards, prolonged stress and a mineral imbalance enhanced exosomes release, which shifted VSMCs toward the calcifying state [38].

#### Inflammatory cell-derived exosomes in atherosclerosis

Inflammation is the core of atherosclerosis, and is present throughout all stages of atherosclerosis. During the early stage, dysfunctional endothelial cells expressing vascular cell adhesion molecule 1 (VCAM-1) trigger adherence of leukocytes, recruitment of monocytes and the migration of platelets towards endothelium [39]. The monocytes differentiate into macrophages which expose scavenger receptors under multiple stimulations of inflammatory mediators. By binding and internalizing lipids through scavenger receptors, macrophages transform into foam cells, and finally become fatty-streak lesions [33]. Exosomes derived from the cell types mentioned above have been reported to play an indispensable role in the progress of atherosclerosis.

Recently progress demonstrated that in male coronary artery disease (CAD) patients, leukocytes could secret extracellular vesicles which mainly contain exosomes with higher sex-determining region Y (SRY) gene and protein than that in normal men. The SRY gene-enriched extracellular vesicles contributed to enhanced adherence of monocytes and endothelial cells. In addition, injection of SRY extracellular vesicles into ApoE^−/−^ mice accelerated atherosclerosis, which suggested a potential pathophysiological role of the leukocyte-derived exosomes [40].

Macrophages play a significant role in atherosclerosis. As a component of the innate immune system, macrophages internalize lipids via scavenger receptors and become foam cells, leading to fatty-streak lesions [41]. Recent studies have suggested that macrophage-derived exosomes may regulate the transition of monocyte and formation of foam cells. In cultured human monocyte-like U937 cells, treatment with oxLDL-immune complex induced the secretion of exosomes containing HSP70 and interleukin-1β (IL-1β), which led to U937 cell activation [42]. In cultured human monocytes and monocyte-like THP-1 cells, galectin-3 (Gal-3) was released via exosomal route in response to phorbol myristate acetate (PMA), after which the cells could differentiate into macrophage [43]. Meanwhile, the released Gal-3 could further promote VSMC differentiation towards osteogenic state and induce atherosclerosis [44, 45]. The above *in-vitro* studies suggested a potential role of exosomes in manipulating the function of macrophage. In addition, macrophage was reported to suppress the migration of endothelial cell through exosomal integrin-1 trafficking [46]. Furthermore, exosomes were involved in macrophage-regulated oxidative stress in atherosclerosis. In monocytes and macrophages, exosomal Gal-3 and thioredoxin-1/peroxiredoxin-1 (TRX-1/PRDX-1) which are sensors of oxidative stress are released under regulation of reactive oxygen species/NAPDH oxidase activity. In other words, the cellular redox status dominated the release of exosomal Gal-3 and TRX-1/PRDX-1. The expression of Gal-3 and TRX-1 in circulation was closely associated with atherosclerosis severity [43, 47]. Latterly, macrophage-derived matrix vesicles were proposed to contribute directly to the microcalcification. The early calcification of atherosclerotic plaques was associated with macrophage accumulation. One study employing *in-vitro* calcifying model revealed that macrophages release matrix vesicles with high calcification and aggregation potential. The work further confirmed that the matrix vesicles not only express exosomal markers—CD9 and TSG101, but also are enriched in S100A9 and annexin V, which contribute to accelerated microcalcification [48, 49].

Within the progress of atherosclerosis, T cells are activated. In advanced human atherosclerotic lesions, activated CD4+ T cells mainly colocalize with marcophages and monocytes [39], and are accounted for 10% to 20% in the lesions’ cell population [50]. The activated T cells augment inflammatory cascade response and profoundly alter macrophage transformation into foam cells [33]. *In-vitro* study has discovered that the exosomes derived from activated human CD4+ T cells are enriched in cholesterol. These derived exosomes were able to enhance cholesterol accumulation and TNF-α production in cultured human monocytes through phosphatidylserine receptor signaling pathway [51]. In addition, exosomes derived from dendritic cells also took part in the activation of CD4+ T cells, and thus, participated in the initiation of adaptive immunity [52].

Mast cells have been observed in atherosclerotic lesions, particularly in fatty streaks [53]. They were influenced by the adjacent activated T lymphocytes and consistently expressed a potent procoagulant factor named plasminogen activator inhibitor type 1 (PAI-1). Al-Nedawi et al. have clarified that mast cell-derived exosomes containing PAI-1 triggered significant upregulation of PAI-1 secretion from endothelial cells. This secretion was believed relevant to endothelial cell dysfunction, resulting in procoagulant states eventually [54].

The adhesion and activation of platelet are critical in the advanced atherosclerotic plaque during atherothrombosis. Platelet contains a number of preformed, morphologically distinguishable storage granules, such as α-granules, dense granules, and lysosomes [55]. Activated-platelet could secret two distinct particles—exosomes and microparticles [56]. While the microparticles have been reported to participate in atherothrombosis and vascular inflammation [57, 58], the mechanism of exosomes is not well established so far. Srikanthan et al. reported that platelet-derived exosomes from human plasma suppressed aggregation of *ex-vivo* platelet and reduced adhesion of platelet to microfluidic flow, suggesting an anti-thrombogenesis effect of exosomes *in vitro*. As a supplementary, *in-vivo* mouse model of FeCl_3_-damaged carotid arteries confirmed the anti-thrombosis effect by exosomes transfusion. The study further elucidated that these platelet-derived exosomes were enriched in ubiquitinated proteins. By increasing the ubiquitination and proteasome degradation of CD36, these exosomes could reduce CD36-dependent lipid loading of macrophages [59]. Moreover, the newly identified miRNome of exosomes derived from human platelets showed that miR-328 and miR-223 were most highly expressed [60]. The latter miR-223 could promote the apoptosis of endothelial cell through insulin-like growth factor 1 receptor, the effect of which was down regulated in response to antiplatelet therapy in symptomatic atherosclerotic patients [61, 62]. Withal, the platelet-regulated exosomal trafficking still needs more investigation.

### Neointima formation and vascular repair

Appropriate neointima formation plays a fundamental role in vascular repair after injury, whereas, excessive neointima formation attributes to the paradoxical restenosis such as restenosis after stenting in stenotic vessels. However, application of exosomes in neointima formation and vascular repair has rarely been reported. The current studies mainly concentrated on exosomes derived from endothelial progenitor cells (EPCs) [63]. In the rat model of balloon-induced vascular injury, exosomes isolated from EPCs accelerated the process of re-endothelialization *in vivo* and enhanced endothelial function *in vitro* [64]. Noteworthy, Spinetti et al. validated that the negative regulators—exosomal miR-15 and miR-16 from human blood, impaired the function of early EPCs in critical limb ischemia patients. Both of two miRs were positively correlated with restenosis after post-revascularization of critical limb ischemia patients [65]. Conceivably, the miRNome and other contents in exosomes may show diverse effects on vascular remodeling and also show a perspective prognostic potential in arterial injury. However, the origins of these circulating exosomes remain unknown, which needs further investigations.

### Primary hypertension

In hypertension, arteries undergo vascular remodeling with increased rigidity and decreased compliance of vessels. The regulation of neuroendocrine system, inflammation and oxidative stress are closely interrelated with each other during the progress of hypertension [66].

Activation of rennin-angiotensin-aldosterone system (RAAS) is the bedrock in hypertension. The end product Angiotensin II of RAS not only regulates vascular function like contraction, growth and fibrosis, but also contributes to increased vascular permeability by initiating inflammatory responses [67]. Pironti et al. discovered that under cardiac pressure overload, cardiomyocytes released exosomes with a key receptor in RAAS inside—the angiotensin II type I receptor (AT1R). The AT1R-enriched exosomes acted on cardiomyocytes, skeletal myocytes, and smooth muscle cells from mesenteric resistance vessels. By improving their sensitivity to RAS, the cells mentioned above could finally adapt themselves to the fluctuation of blood pressure [68]. Moreover, it highlighted the modulatory role of AT1R-enriched exosomes in vascular responses to neurohormonal stimulation. In addition, during the chronic inflammation in hypertension, macrophages were assumed to damage endothelial cell function through exosomal pathway. Since exosomes derived from serum of hypertensive rats or from activated macrophages showed same signal transduction pathway such as p38 MAPK and ICAM-1 in human coronary arterial endothelial cells (HCAECs), endothelial damage in hypertension may be partially associated with macrophage-derived exosomes [69].

However, differing from the limited publications in the preclinical research of exosome in hypertension, there are relatively more researches about the urinary exosomes for diagnose in hypertension. The urinary exosomes secreted by multiple cell types in kidney could be easily detected, and the main exosomal protein- sodium transporter is correlated with hypertension [70]. Recent studies have confirmed that urinary exosomal content is altered by RAAS activation [71]. The levels of exosomal sodium transporters including sodium chloride cotransporter, alpha- and gamma-epithelial sodium channels were strongly correlated with the progression of hypertension [71–75]. The miRNome analysis of urinary exosomes from hypertensive patients also proved that several miRNAs like miR-615, miR-211 were sensitive to fluctuation of blood pressure [76]. Overall, the studies on the content of urinary exosomes exhibited a promising prognostic application on hypertension in the future.

### Pulmonary arterial hypertension

Pulmonary vascular remodeling is a key pathological feature of pulmonary arterial hypertension (PAH). The pathogenesis is characterized by increased proliferation and apoptosis resistance of pulmonary endothelial cells and VSMC. The alternations of EC and VSMC lead to vascular thickening and stiffening, which results consecutively in an imbalanced hemodynamics with low flow and high resistance [77]. Emerging evidences have revealed that exosomes derived from the involved cell types control the underlying changes of PAH.

Increasing articles have discovered a crucial exosomal trafficking between pulmonary VSMCs and endothelial cells in PAH. In chronic hypoxia pulmonary hypertension mouse model, exosomes secreted by pulmonary VSMCs were enriched with miR-143. These exosomes could be transported to pulmonary endothelial cells, inducing migration and angiogenesis. Genetic ablation and pharmacological inhibition of miR-143 prevented the development of PAH. A reduction in microvessel density was also observed in the miR-143^−/−^ mouse, indicating that transportation of miR-143 into exosomes could enhance microvessel density [78]. This study identified a critical exosome-mediated cell-to-cell communication in the pathogenesis of PAH. The functions of exosomes are determined by its cell type-specific molecular composition. For instance, the mesenchymal stromal cell (MSC)-derived exosomes, exhibited a cytoprotective action in pulmonary hypertension. In mouse hypoxic pulmonary hypertension model, intravenous delivery of MSC-derived exosomes inhibited vascular remodeling and alleviated pulmonary hypertension through the suppression of signal transducer and activator of transcription 3 (STAT3) and the upregulation of the miR-17 superfamily. *In-vitro* study also confirmed the anti-inflammatory role of these exosomes and their direct inhibitory effect of STAT3 signaling on pulmonary endothelial cells [79]. Hence, exosomes may also be implied as a promising therapeutic potential in lung injury.

### Aortic aneurysm

Expansion and rupture of aortic aneurysm (AA) including abdominal aortic aneurysm (AAA), thoracic aortic aneurysm, and intracranial aneurysm is now one of the leading death causes all over the world. AA shares similar pathogenesis with other vascular diseases. The pathogenesis of AA involves in two major aspects: the degradation of the extracellular matrix which weakens the aortic wall; the inflammatory infiltrates within the wall of aortic aneurysms which accelerates aneurysm progression. Moreover, genetic, environmental and hemodynamic factors all contribute to the development of AA [80, 81].

VSMCs and macrophages are the main origin of matrix metalloproteinases (MMP) which is capable of degrading extracellular matrix. Later on, platelets could also take part in the thrombosis [80]. Previous work mainly focused on the inflammatory regulation among the involved cells in AA. Until recently are studies regarding the role of exosomes in AA just appearing. Martinez-Pinna et al. isolated exosomes and microparticles from plasma of AAA patients and then analyzed the protein profiles by applying a label-free quantitative MS-based strategy. As a result, exosomes from AAA patients contained much higher platelet factor-4 (PF-4), ferritin light chain, HSP60 and C-reactive protein than those of controls [82]. PF-4, an important chemokine triggering leucocyte recruitment to AAA, was released from activated platelets, which suggested the PF-4-enriched exosomes may be derived from platelets [83]. Meanwhile, ferritin light chain and HSP60 have been involved in the regulation of oxidative stress in AAA, which implied a potential pathogenetic role of exosomes in AAA [84, 85]. Furthermore, exosomes also exhibited a therapeutic potential in AA. Previous studies have discovered that bone marrow-derived MSCs could reduce the incidence rate of aortic aneurysm [86, 87]. *In-vitro* experiment further verified that exosomes derived from MSCs exhibited anti-inflammatory effect on macrophages and aortic smooth muscle cells, which suggested that exosomes might also be a novel therapeutic tool for AA [88].

## TRANSLATIONAL APPLICATIONS TARGETING EXOSOME

Exosomes exist in all body fluids. It can be isolated by the “golden method” named “differential ultracentrifugation and density gradient centrifugation” [89]. Benefited from the structure of lipid bilayer, the exosomal content could be remained steadily rather than digested by various circulatory enzymes. Meanwhile, the content in exosome is largely determined by the pathophysiological status of cells or tissues. Hence, the exosomal content exhibit the prognostic potential in various diseases, for example, the sodium chloride cotransporters could be a potential biomarker for hypertension [71].

Furthermore, since exosomes possess some properties such as biocompatibility, biological barrier permeability, low toxicity and low immunogenicity, which are good for therapeutic delivery [90, 91]. Exogenous miRNAs, siRNAs, and even drugs could be encapsulated into the naïve exosomes or engineered exosomes. The proven technology that packages the therapeutic miRNAs and siRNAs into engineered exosomes could be achieved by the following means: 1) co-transfection into the donor cells with one plasmid or virus encoding the precursor miRNAs or siRNAs, and the other plasmid encoding fusion targeting cassette, respectively [90]; 2) electroporation of synthetic miRNAs or siRNAs into purified exosomes directly [92, 93]; 3) transient transfection of miRNAs by using commercial available transfection reagents [94]. In addition, permeabilization with soponin, sonication, or extrusion could increase the loading efficiency [95]. Besides, the technology of assembling drugs into exosomes has been gradually developed. Three distinct approaches are utilized for the drug loading: 1) direct incorporation of drugs into purified exosomes such as lipophilic small molecules, low molecular antioxidant and anticancer agents [92, 96, 97]; 2) drugs are loaded into donor cells and ultimately equipped into exosomes following exosomal release [95, 98]; 3) drug-encoding DNA are directly transfected into donor cells, which results in drug expression and sorting into exosomes [98, 99].

So far the vast majority of these methods were applied in oncology. For instance, extracellular vesicles delivering siRNA that targeted the oncomiR-miR-150 neutralized the proangiogenic effect of miR-150 in mouse xenograft tumor model of S-180 sarcoma cells and attenuated tumor angiogenesis [100]. Moreover, a limited number of early-phase clinical trials about the therapeutic potent of exosomes in cancer have been undertaken over the past decades, showing potential safety and feasibility of exosomal therapy [101]. For instance, Morse MA et al. have tested the safety, feasibility and efficacy of autologous dendritic cell-derived exosomes (DEX) loaded with the MAGE tumor antigens in patients with non-small cell lung cancer (NSCLC). The patients with advanced NSCLC showed good tolerance to the DEX therapy, some of whom even experienced long term stability of disease and activation of immune effectors [102]. Another study enrolling 40 patients with advanced colorectal cancer also revealed that the immunotherapy of colorectal cancer with autologous ascites-derived exosomes loading GM-CSF was feasible and safe [103].

However, the application in cardiology is limited. Recently, a study using curcumin-primed exosomes isolated from curcumin transfected donor cells confirmed that curcumin could mitigate endothelial cell dysfunction in hyperhomocysteinemia, a high risk factor of coronary artery disease [104]. Overall, however, some technological, functional and safety features of exosomal engineering are yet to be addressed. The molecular mechanism of exosomal biogenesis still needs further exploration.

## CONCLUSIONS

Increasing numbers of studies highlight the contribution of exosomes in the intercellular communication in vascular remodeling. As an alternative carrier, exosomes internalize contents based on the patho/physiological status, inducing various effects subsequently. However, there are still many issues to be resolved regarding the mechanisms of exosomal biology such as exosome formation, release, internalization and clearance. Besides, despite interesting promises for the prognostic and therapeutic approaches in vascular diseases, exosome-based therapies are still limited and require further preclinical studies before translational applications.
